# Colorectal cancer and therapy response: a focus on the main mechanisms involved

**DOI:** 10.3389/fonc.2023.1208140

**Published:** 2023-07-19

**Authors:** Sara Tirendi, Barbara Marengo, Cinzia Domenicotti, Anna M. Bassi, Vanessa Almonti, Stefania Vernazza

**Affiliations:** ^1^ Department of Experimental Medicine, University of Genoa, Genoa, Italy; ^2^ Inter-University Center for the Promotion of the 3Rs Principles in Teaching & Research (Centro 3R), Genoa, Italy

**Keywords:** CRC, adjuvant treatments, chemoresistance, CSCs, drug delivery system

## Abstract

**Introduction:**

The latest GLOBOCAN 2021 reports that colorectal cancer (CRC) is the second leading cause of cancer-related death worldwide. Most CRC cases are sporadic and associated with several risk factors, including lifestyle habits, gut dysbiosis, chronic inflammation, and oxidative stress.

**Aim:**

To summarize the biology of CRC and discuss current therapeutic interventions designed to counteract CRC development and to overcome chemoresistance.

**Methods:**

Literature searches were conducted using PubMed and focusing the attention on the keywords such as “Current treatment of CRC” or “chemoresistance and CRC” or “oxidative stress and CRC” or “novel drug delivery approaches in cancer” or “immunotherapy in CRC” or “gut microbiota in CRC” or “systematic review and meta-analysis of randomized controlled trials” or “CSCs and CRC”. The citations included in the search ranged from September 1988 to December 2022. An additional search was carried out using the clinical trial database.

**Results:**

Rounds of adjuvant therapies, including radiotherapy, chemotherapy, and immunotherapy are commonly planned to reduce cancer recurrence after surgery (stage II and stage III CRC patients) and to improve overall survival (stage IV). 5-fluorouracil-based chemotherapy in combination with other cytotoxic drugs, is the mainstay to treat CRC. However, the onset of the inherent or acquired resistance and the presence of chemoresistant cancer stem cells drastically reduce the efficacy. On the other hand, the genetic-molecular heterogeneity of CRC often precludes also the efficacy of new therapeutic approaches such as immunotherapies. Therefore, the CRC complexity made of natural or acquired multidrug resistance has made it necessary the search for new druggable targets and new delivery systems.

**Conclusion:**

Further knowledge of the underlying CRC mechanisms and a comprehensive overview of current therapeutic opportunities can provide the basis for identifying pharmacological and biological barriers that render therapies ineffective and for identifying new potential biomarkers and therapeutic targets for advanced and aggressive CRC.

## 1 Introduction

Colorectal cancer (CRC) is the third most diagnosed cancer and the second leading cause of death worldwide regardless of gender (1). Approximately 90% of CRCs are adenocarcinoma originating from epithelial cells of the colorectal mucosa, whilst the remaining 10% are represented by rare CRC types (i.e., squamous cell carcinoma, adenosquamous carcinoma, spindle cell carcinoma, and undifferentiated carcinoma) (2).

Most CRC cases are sporadically and associated with non-hereditary spontaneous mutations and epigenetic aberrations arising from several risk factors, including dysregulation of the gut microbiome, obesity, sedentary lifestyle, excess intake of meats, fats, starches, and sugars, folate deficiency, alcohol, cigarette smoking, and so on (3). However, a lower percentage of cases (about 30%) is represented by familial cases, of which approximately 5% present specific genetic signatures, penetrance, and transmission due to germline variants in CRC predisposing genes, e.g., adenomatous polyposis coli (APC), mismatch repair (MMR) genes, epithelial cell adhesion molecule (EPCAM), SMAD4/BMPR1A, and MUTYH (4–7).

Data report that the highest CRC incidence rates are recorded in developed countries and the incidence of early-onset CRC in individuals younger than 50 continues to rise (2, 4). Therefore, to facilitate diagnosis of CRC cancer in earlier stages, the recommended screening age was recently lowered to 45.

Early-stage colon cancer may be asymptomatic and often become symptomatic late in the disease. Indeed, about >25% of patients are diagnosed with advanced disease, i.e., extensive or metastatic colorectal cancer (mCRC), at the time of diagnosis, while more than 50% of patients with the initially localized disease develop metastases during or after therapies (8, 9). As known, metastasis poses a huge clinical challenge because only 20% of mCRC patients survive (10).

When neoadjuvant therapy is not included in the treatment plan, surgical resection is performed as the first curative intent in patients with localized and locoregional CRC (stages I, II, and III), as well as for those with resectable distant metastases (5, 11–13). However, the National Comprehensive Cancer Network (NCCN) guidelines recommend neoadjuvant oxaliplatin-based chemotherapy for patients with “bulky nodal disease or clinical T4b” colon cancer to decrease the size of the tumor before surgery (14). To reduce the risk of cancer recurrence and improve patient outcomes, an adjuvant postoperative chemotherapy regimen is routinely employed in stage III patients (i.e., localized tumor with lymph node invasion) and, in some cases, in stage II patients (i.e., localized tumor w/o lymph node invasion) (15, 16). Moreover, chemotherapy is the first-line therapy also for mCRC treatment.

The genetic variability of CRC makes necessary to identify the tumor subtypes (e.g., mismatch repair or microsatellite instability status, mutations in KRAS, NRAS, BRAF) to set the most suitable adjuvant therapy (i.e., systemic chemotherapy alone or with other FDA-approved drugs).

The CRC prognosis depends essentially on comorbid conditions, the frailty of patients, and drug resistance promoted by cancer stem cells and/or genetic mutations in key driver genes (e.g., KRAS, p53, BRAF) (17, 18). Therefore, this present review aims to summarize the mechanisms that characterize the stepwise nature of CRC, its genetic landscape, and the current and future approaches for CRC management.

## 2 Methods

The data in this present systematic review were collected using two different searches: PubMed (https://pubmed.ncbi.nlm.nih.gov/) and an online bioinformatic database (http://clinicaltrials.gov). The search of the references using PubMed identified a total of 144 hits from 1988 to 2022 relatively to keywords such as “Current treatment of CRC” or “immunotherapy in CRC” or “gut microbiota in CRC” or “chemoresistance and CRC” or “oxidative stress and CRC” or “novel drug delivery approaches in cancer” or “CSCs and CRC” or “systematic review and meta-analysis of randomized controlled trials”. Thus, the combined information obtained from the two data sources has represented the basis for writing the review.

## 3 Results

### 3.1 Mechanisms of CRC initiation and progression

**Figure 1:**
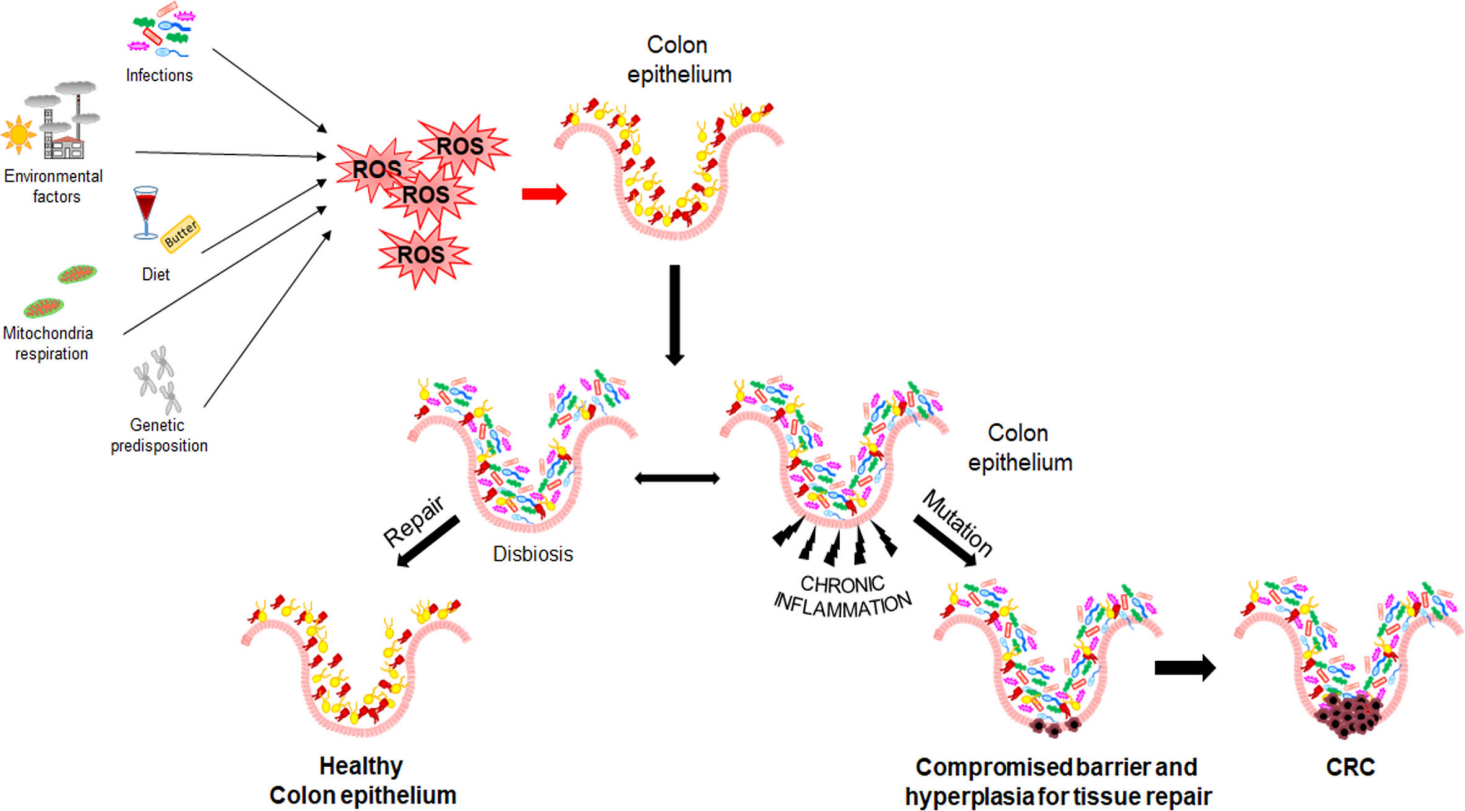
Mechanisms of CRC initiation and progression. Environmental and genetic risk factors promoting Reactive Oxygen Species production, dysbiosis, and inflammation.

The stepwise nature of sporadic CRC is still poorly understood, even though several mechanisms have been described to be involved in its initiation and progression. Epidemiological studies have found a relationship between CRC and chronic exposure to environmental risk factors (see above section) with strong pro-inflammatory potential. Moreover, increasing evidence suggests that intestinal microbiota and its products (e.g., butyrate and bacterial toxins) play a pivotal role in all CRC steps (initiation, progression, and metastasis) (19–21) (**Figure 1**). CRC patients display a reduced bacterial diversity and richness compared to healthy individuals, reflecting a distinctive intestinal microbial dysbiosis (22). Dysbiosis causes alteration in gut mucosa integrity and permeability, due to alteration of intercellular tight junctions. This condition, enhancing the colocyte susceptibility to mutagenic/carcinogenic factors and pathogenic bacteria, also promotes the activation of Mucosal Associated Lymphatic Tissue. Moreover, Th2-derived cytokines induced by pathogens or autoantigens may result in myeloid cell recruitment (neutrophils and macrophages) and, consequently in Reactive Oxygen Species (ROS) production.

As known, inflammation and oxidative stress are tightly coupled; in fact, a chronic activation of inflammatory cells and production of pro-inflammatory mediators (e.g., cyclooxygenase 2, prostaglandin E2, tumor necrosis factor α, and transforming growth factor β) enhance ROS generation and dysregulate the activity of signal transduction pathways, including Transducer and Activator of Transcription 3, Nuclear Factor-kappa B (NF-kB), hypoxia-inducible factor-1α (HIF1α), NF-E2 related factor-2 (NRF2) (23–25). In addition, it has been reported that ROS overproduction may result in genetic/epigenetic changes, such as single-strand cleavage, point mutations, miscoding, abnormal amplification, oncogene activation, and immune suppression, leading to possible precancerous lesions (i.e., adenomatous polyps) (26, 27).

**Figure 2:**
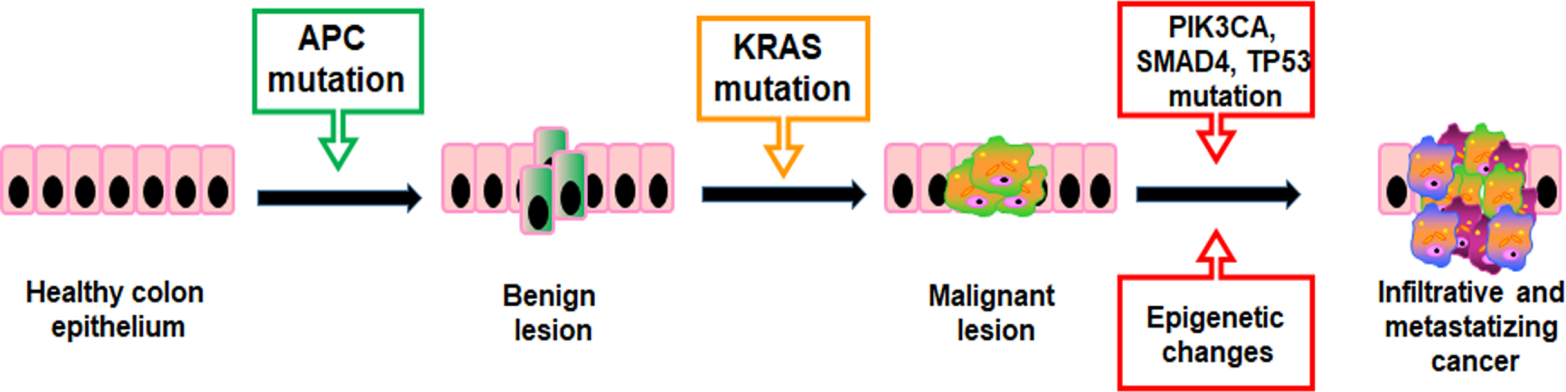
Stepwise molecular/genetic events that underlie the initiation and progression of CRC.

Mutations providing a selective growth advantage to the cells within their microenvironment can potentially drive cancer development. Generally, two mutated gene drivers can lead to a net cell gain and detectable benign lesions, but over three gene mutations promote the invasion through the basement membrane, thereby leading to malignancy. In CRC, the first mutations usually concern the APC gene resulting in a proliferative advantage of epithelial cells promoting benign lesions (small adenomas) (**Figure 2**). APC mutated small adenomas have a slow growth rate, but further mutations of the KRAS gene can increase their proliferation. However, the mutational status may be worsen by sequential mutations of genes such as PIK3CA, SMAD4, and TP53 that promote the onset of malignant tumors capable of infiltrating surrounding tissue and metastasizing distant organs (**Figure 2**) (28). In addition, both colorectal adenomas and CRC are linked to epigenetic alterations such as aberrant DNA methylation in key tumor-suppressor and oncogenes and dysregulation of miRNA expression (29, 30).

Well-known examples of the carcinogenic role of ROS in CRC are missense mutations at p53 suppressor gene, activation of canonical Wnt signaling pathway (Wnt/β-catenin), which is involved in cancer stem cell renewal process, and PI3K/Akt signaling pathway, which regulates cell proliferation (31, 32). Moreover, the most frequent ROS-dependent pre-mutagenic DNA lesion is represented by 8-oxoguanine (8-oxoG) (33).

Notably, oxidative stress, due to either ROS overproduction or a reduced activity of the enzymatic and non-enzymatic antioxidant systems, is often involved in development and progression of several cancers via activation of redox-responsive signaling pathways leading to uncontrolled cell growth and oxidation of lipids, carbohydrates, and proteins (i.e., cancer initiation, promotion, and progression stages). Accordingly, oxidative stress is one of the main cancer research topics given its involvement in both genetic and metabolic cell damage (34–36). Increasing evidence showed that oxidative stress control biogenesis of cancer-associated microRNA (miRNA) via targeting various transcription and epigenetic factors. Recently, CRC-associated miRNAs (*e.g.*, miR-106b-5p, miR-335-5p, miR-193a-5p, miR-378a-3p and miR-423-5p) are becoming attractive biomarkers as they are expressed from the early stage of tumor development (37).

Moreover, either up-regulation or down-regulation of miRNAs also known as “onco-miRNAs” are involved in CRC progression and metastasis contributing to dysregulate several signaling pathways, including mitogen-activated protein kinases (e.g., miR-422a, miR-195), Wnt (e.g., miR-135a, miR-135b, miR-155, miR-17–5p, miR-224), transforming growth factor-β (e.g., miR-224, miR-20a-5p) and epithelial-to-mesenchymal transition (EMT) (e.g., miRNA-155, miR-34) (38–40).

Oncogene, oncosuppressor and metabolic gene mutations contribute to the profound metabolic alterations found in cancer cells, i.e., impaired respiration, increased fermentation and anabolism (41). In most cases, the metabolism of cancer cells favors aerobic glycolysis (the Warburg effect) rather than oxidative metabolism to fulfill their biosynthetic and bioenergetic demands of rapid and sustained proliferation. Mitochondrial Oxidative Phosphorylation System (OXPHOS) is not necessarily defective in tumorigenic cells, and it can take place proportionally to the oxygen supply. Indeed, it has been shown that cancer stem cells are able to revert glycolysis to TCA cycle to better satisfy their metabolic needs and overproduce ROS (42). The Warburg phenotype has been demonstrated to be driven by overexpression of oncogenes such as c-Myc and HIF-1α (43). The inhibition of pyruvate dehydrogenase activity and the increase of lactate dehydrogenase activity lead to the conversion of pyruvate to lactate following the mass action law (44). Moreover, lactate is also generated from catabolism of glutamine and it is considered a metabolite eliciting a broad spectrum of effects useful to sustain cancer progression and metastasis (45). Cancer cells are capable of adapting to metabolic-derived acidosis via monocarboxylate transporters (MCTs), which export lactate and favor intracellular alkalinization. Thus, the lactate exported by tumor cells can be imported by cells of tumor microenvironmental where it acts as important intracellular signaling for angiogenesis (46, 47).

Notably, cancer cells well adapt to ROS by triggering a powerful antioxidant response mainly driven by glutathione (GSH) and antioxidant enzymes, such as superoxide dismutase, catalase, peroxiredoxins, GSH peroxidases, and thioredoxins (48). Thus, the maintenance of the oxidative balance enables cancer cells to perform their biological functions such as proliferation, differentiation, and migration (49–51).

#### 3.1.1 Genetic- molecular heterogeneity of CRC

CRC from a genetic-molecular standpoint is extremely diversified. In fact, there are four main mechanisms of gene alteration: (i) microsatellite instability (MSI), (ii) chromosomal instability (CIN), (iii) CpG island methylator phenotype (CIMP), (iv) and BRAF or KRAS mutations (52). Another aspect concerning the molecular and phenotypic differences is the tumor localization (i.e., the right or left side), which leads to different gene expression and mutation profiles. Right-sided CRC occurs mainly in patients with genetic predisposition and is characterized by hypermethylation, higher frequency of BRAF mutation, and, in some cases, MSI (53). Instead, left-sided CRC is characterized by CIN and the activation of the EGFR pathway (54). Moreover, differences in tumor microenvironment components (e.g., tumor epithelial cells, immune cells, and cancer-associated fibroblasts) play a critical role in defining CRC with a positive or poor prognosis and in maintaining immune surveillance (through the increase in tumor T-lymphocyte subset density) or in promoting immune escape (55). For example, a high density of specific tumor-infiltrating lymphocytes (i.e. cytotoxic and memory T-cells) in MSI-high CRC can be considered a favorable prognostic marker, because it counteracts the establishment of the “immunoediting” process and reduces the tumor spread (56–58). Furthermore, Canna et al. (59), found a relationship between systemic inflammatory response and local inflammatory response in patients undergoing resection for CRC, demonstrating that a high concentration of C-reactive protein and low tumor-infiltrating CD4^+^ are predictive of poor cancer-specific survival.

The analysis of genetic profiles cannot be used for clinical purposes due to a discrepancy in results (e.g., sample preparation methods, use of different data processing and algorithms among different patient cohorts, gene expression platforms, and so on). However, the consensus molecular subtypes (CMS), which represent a transcriptome-based classification of CRC, include some superficial similarities useful for predictable CRC prognosis (60, 61). The first called CMS1 (MSI immune) is characterized by hypermutation, frequent BRAF mutation, MSI, and strong immune activation, the CMS2 (canonical) by CIN and marked WNT and MYC signaling, and the CM3 (metabolic) by evident metabolic dysregulation and KRAS-mutated tumors. Lastly, the CM4 (mesenchymal), includes tumors characterized by prominent TGFβ activation, epithelial-mesenchymal transition gene up-regulation, angiogenesis, and matrix remodeling.

### 3.2 Adjuvant treatments of CRC

Chemotherapy agents are usually used after a surgical excision as the treatment of choice to eradicate Minimal Residual Disease (MRD) in high-risk stage II and stage III patients and to increase the overall survival rate in stage IV patients (62–65). However, the only use of chemotherapy as standard-of-care (**Table 1**) can represent a limit due to the high systemic toxicity, unsatisfactory response rate, the onset of drug resistance, and the low tumor-specific selectivity. Therefore, massive investments have been earmarked to develop new approaches to improve patient outcomes. The identification of point mutations in specific oncogenes (KRAS, NRAS, and BRAF), amplification of human epidermal growth factor receptor 2 (HER2), the MSI status, the DNA mismatch repair status (deficiency or proficiency), has provided a framework for finding additional approaches, as well as new prognostic perspective (66). Up to today it is possible to hit the cancer more effectively, by administering the most suitable biological agents with the standard chemotherapy taking into account the genetic setting of patients (67, 68). In this regard, targeted therapies (i.e., antibodies and small molecules) and immunotherapies, which actively or passively target the patient’s immune system, are widely used in combination with FOLFOX or FOLFIRI as a first-/second-line setting or alone as a third-line setting to improve the overall survival (OS) and progression-free survival (PFS) of advanced/metastatic cancer patients (69, 70). Moreover, antitumor immunity exerted by vaccines, specialized dendritic cells or new generation of cytotoxic T cells are currently under investigation in clinical trials (71, 72) (**Table 2**).

**Table 1:** The main therapeutic approaches in the CRC treatment.

Cytotoxic drug regimen	• Fluoropyrimidines; • FOLFOX (5-FU/LV/Oxaliplatin); • FOLFIRI (5-FU/LV/Irinotecan).
Targeted and immune-therapies	• EGFR inhibitors; • Anti-angiogenesis therapies; • BRAF inhibitors; • Kinase inhibitor; • Immunotherapeutics • HER2 inhibitors; • KRAS inhibitors.

**Table 2:** Current clinical trials based on immunotherapy.

Clinical trials N°	CRC stage	Treatment	Stage of trials	Status of trials
NCT01890213	III	CEA (6D) VRP vaccine	I	Completed
NCT02466906	III	RhGD-CSF	II	Unknown
NCT02912559	III	Chemotherapy and Atezolizumab	III	Active
NCT02280278	Post-therapy III	Cytokine-induced Killer cell Immunotherapy	III	Unknown
NCT03507699	Metastatic	Nivolumab, Ipilimumab CMP-001 and radiosurgery	I	Completed
NCT04044430	Metastatic	Encorafenib, Binimetinib and Nivolumab	I	Completed
NCT05130060	Metastatic	Vaccine (PolyPEPI1018) and TAS-102	I	Active
NCT03310008	Metastatic	NKR-2 + Folfox	I	Unknown
NCT02834052	Metastatic	Pembrolizumab and Poly-ICLC	I/II	Completed
NCT03377361	Metastatic	Nivolumab, Trametinib with or without Ipilimumab	I/II	Active
NCT03436563	Metastatic	Anti-PD-L1/TGFβII fusion protein M7824	I/II	Active
NCT03711058	Metastatic	Copanlisib and Anti-PD1 Nivolumab	I/II	Active
NCT04599140	Metastatic	CXCR1/2 inhibitor (SX-682) and Nivolumab	I/II	Recruiting
NCT03993626	Metastatic	CXD101 and Nivolumab	I/II	Unknown
NCT02981524	Metastatic	GVAX (with CY) colon vaccine and Pembrolizumab	II	Completed
NCT04109924	Pre-treated metastatic	TAS-102 Irinotecan and Bevacizumab	II	Active
NCT04362839	Chemoresistant metastatic	Regorafenib, Ipilimumab and Nivolumab	I	Active

In patients with left-sided KRAS wild-type tumors, for instance, the administration of anti-EGFR (i.e., cetuximab or panitumumab) in combination with standard-of-care chemotherapy as a first-line setting shows improvement in both OS and progression-free survival (PFS) (73–75). Additionally, anti-EGFR can be used alone in chemo-refractory patients with advanced CRC (76).

Recently, in patients with BRAF-mutated mCRC, the use of anti-EGFR in combination with a selective inhibitor of BRAF kinase (encorafenib) and a reversible inhibitor of the kinase activity of mitogen-activated extracellular signal-regulated kinase 1 (MEK1) and MEK2 (binimetinib) has been proposed as the third line treatment to improve the prognosis (77, 78).

Among the first- and second line interventions for mCRC, also the VEGF inhibitors (i.e., Bevacizumab, Aflibercept) in combination with standard-of-care chemotherapy contribute to improve OS and PFS in patients (79, 80).

Another targeted therapy to treat advanced/metastatic CRC refractory to all standard treatments is represented by the diphenylurea-based multikinase inhibitor (i.e., Regorafenib). This anti-tumoral drug targeting multiple protein kinases regulating angiogenesis, proliferation, immunity, and metastases, increases OS of heavily pre-treated patients (79, 81–83).

KRAS-targeted drugs, such as sotorasib and adagrasib, are emerging for their anti-cancer activity in heavily pre-treated patients harboring the KRAS^G12C^ mutation. These drugs are small molecules that keep KRAS in its inactive state, allowing apoptosis. However, their use in CRC treatment is still under investigation (84).

#### 3.2.1 5- fluorouracil

5-FU is a fluorinated analogue of uracil that belongs to fluoropyrimidines. It was developed in 1957 and still today, it represents the mainstay of systemic combination chemotherapy for the treatment of CRC (85, 86). Although several 5-FU administration schedules were used (e.g., bolus intravenous, bolus *plus* intermittent intravenous infusion), today the standard of care is represented by continuous or intermittent intravenous infusion (87). Moreover, for around twenty years, also oral 5-FU prodrugs (*e.g.*, Capecitabine, Tegafur, 5′-deoxy-5-fluorouridine) are commonly used as part of combination regimens or as monotherapy (88, 89).

5-FU is easily incorporated into DNA and RNA where it acts as an antimetabolite because shares a common structure with pyrimidines (90). After administration, 5-FU is converted via anabolic pathways into fluorodeoxyuridine monophosphate (FdUMP), fluorodeoxyuridine triphosphate (FdUTP), and fluorouridine triphosphate (FUTP) (91). Stable complex between FdUMP and thymidylate synthase (TS) inhibits deoxythymidine mono-phosphate production, which consequently results in severe disruption of DNA synthesis and repair. Leucovorin (LV, Folinic acid), and Methotrexate (MTX, Folate analogue) are preferably used in combination with 5-FU to improve its antitumor activity (92, 93). Moreover, metabolites of 5-FU produce also alterations in the cellular membrane (89).

Approximately 80% of the total 5-FU dose is metabolized primarily in the liver by dihydropyrimidine dehydrogenase (DPD) (94), an enzyme that catalyzes the rate-limiting step in its metabolism.

Severe 5-FU-associated toxicity (e.g., leukopenia, neutropenia, thrombocytopenia, anemia, neuropathy, skin rash, hand-foot syndrome, and so on) is mainly due to a partial or complete DPD deficiency (95–99). In particular, different rare variants in the gene encoding DPD (DPYD) have been identified as validated risk variants for drug toxicity (86). Therefore, FDA-approved drug label prevents the use of 5-FU in individuals with absent DPD activity (88), while the Clinical Pharmacogenetics Implementation Consortium and the Dutch Pharmacogenetics Working Groups report dosing recommendations for 5-FU-based chemotherapy, based on DPYD genotype (88, 100, 101).

Although 5-FU-based chemotherapy combining with oxaliplatin or irinotecan has improved the response rate in patients with advanced CRC, primary or acquired chemoresistance is the leading cause of unsatisfactory outcomes in over 90% of patients with metastatic disease (93, 102). Indeed, intratumoral heterogeneity due to genetic mutations, tumor microenvironment (TME), and the presence of cancer stem cells, and the molecular complexity of CRC as well, making it necessary to develop other novel therapeutic strategies to overcome drug resistance and improve drug response rates.

### 3.3 The presence of cancer stem cells limits therapy efficacy against CRC

The main limiting factor for cancer patients is the onset of multi-drug resistance (MDR), which makes cancer cells tolerant to anti-cancer drugs. In fact, the combined chemotherapy and the development of different administration schedules are not always sufficient to avoid these issues due to the biological complexity of the tumor (103).

Tumor MDR is a highly-complex phenomenon that encompasses a plethora of molecular mechanisms involving not only cancer cells, but also infiltrating cells (e.g., endothelial, hematopoietic, and stromal cells) and the resulting tumor microenvironment (104). The constant interactions between tumor cells and their surrounding stroma result in alterations of many different cellular processes. Moreover, the presence of a sub clonal variation among cancer cells allows greater adaptability of the tumor to therapy, promoting its evolution.

Multiple molecular mechanisms have been identified as contributing factors to MDR development. Among these, the interplay between pre-existing and drug-induced mechanisms, including defects in the apoptotic machinery, mitochondrial dysfunction, altered autophagy activity, aberrant cell signaling, reduction in drug concentration and genetic and epigenetic changes, plays a significant role (105–108).

Moreover, the major cause of primary therapy resistance is represented by unresponsive subpopulations, such as cancer stem cells (CSCs) that can increase by up to 30% following long-term drug treatment (109).

Stochastically CSCs are distributed within tumors, but preferably they reside in specific niches, characterized by hypoxia, low pH, and fewer nutrients, which in addition to conferring them stemness features, allow the generation of differentiated progenies (110, 111).

CSCs are frequently quiescent and poorly differentiated cell populations with a lower level of intracellular ROS that share with normal stem cells both properties (i.e., self-renewal, self-sufficiency, and differentiation), and stemness signaling pathways (e.g., Notch, Sonic hedgehog, WNT/β-Catenin, JAK/STAT, and NF-kB). Their origin is still debated but it has been suggested that, at the moment of tumor initiation, the acquisition of CSC phenotype from either transformed differentiated cells (stochastic model) or transformed tissue-resident stem cells (hierarchical model) is promoted by the overexpression of oncogenes and the inhibition of tumor suppressor genes (e.g., APC, TP53, TGFBR2, SMAD4, PTEN, and RAS). Instead, following chemotherapy or radiotherapy regimen, new CSCs derive from either non-CSC subpopulations or therapy-induced senescent tumor cells (112).

Standard chemotherapy is not a valid therapeutic option for CSCs because they can effectively counteract the chemotherapy-induced oxidative stress through their free radical scavenging systems, such as GSH, and overexpression of the anti-apoptotic protein B-cell lymphoma 2 (Bcl2) (113–115). Moreover, the enhancement of ATP-binding cassette (ABC) transporters and aldehyde dehydrogenase (ALDH) expression, the increased resistance to apoptosis, and the activation of DNA damage sensor and repair machinery contribute to give to CSCs a survival advantage against anti-cancer therapy (116). Additionally, CSCs can transiently and reversibly switch between epithelial and mesenchymal states and *vice versa* (i.e., epithelial-mesenchymal plasticity) via Wnt/b-catenin signaling (117, 118). Such versatility, consequently, results in metabolic reprogramming in cellular bioenergetics, where energy supply can alternatively depend on aerobic glycolysis or mitochondrial OXPHOS.

It has been shown that metformin potentially offers therapeutic advantages by inhibiting the mitochondrial respiration, forces CSCs to a metabolic shift from OXPHOS to glycolysis (119, 120). The temporary OXPHOS suppression renders CSCs more prone to apoptosis. However, tumor relapse under metformin treatment cannot be excluded since CSCs can acquire resistance mainly due to MYC overexpression, promoting a Warburg-like glycolytic phenotype (120). Also, progressive increase in glycolysis-derived lactate may promote the activation of proteases, leading to ECM degradation, and resistance to chemotherapy (121).

The release of IL-4 from colorectal CSCs promotes their survival and hampers the CD8+T cell-mediated antitumor immune response, while the presence of inflammatory cytokines, including IL-1, IL-4, IL-6, IL-8, IL-10, and TGF-β, fuels an inflammatory loop, via Stat3/NF-κB pathways, for stimulating the self-renewal of CSCs (118, 122). Moreover, the tumorigenic and self-renewal capacity of CSCs also depend on the hyperactivation of β-catenin, Notch, and Hedgehog signaling pathways (123, 124).

Although several CSC biomarkers have been identified for CRC, their preclinical application is still unavailable due to the intrinsic features of CSCs, i.e., phenotypic heterogeneity, and the influence of the TME or CSC behavior. In this regard, previous studies have focused the attention on both the CSC-related signature and immune cell infiltration as important prognostic factors. The correlation between infiltrating portion of immune cells, i.e., tumor immune microenvironment (TIME), and hallmark gene sets may represent a possible starting point for developing CSC-targeted therapeutic strategies (122, 125).

### 3.4 New drug delivery approaches and latest strategies implemented in the treatment of CRC

Increasing evidence suggests that the use of nanoscale nanoparticles (NPs) as drug delivery systems (DDS), including liposomes, nanoemulsions, hydrogels, multifunctional inorganic materials (e.g., carbon nanotubes, gold nanoparticles, quantum dots), and peptides, could provide a novel therapeutic approach useful in overcoming MDR and improving the pharmacokinetics and biodistribution of anticancer compounds, resulting in reduced side effects (126). These NPs refers to nanometer scale systems (10-1000 nm) capable of protecting encapsuled molecules from degradation and passively or actively delivering drugs, small molecules, proteins, peptides, DNA and RNA into specific targets. However, their bio-distribution and clearance in the body depend not only by the NP chemical, physical and biological properties (*e.g.*, size, stability, surface charge, solubility, and so on) but also by factors, such as the administration route (e.g., intravenous, oral, pulmonary and dermal administration) and host environment (e.g., pre-existing inflammation) (127).

**Figure 3:**
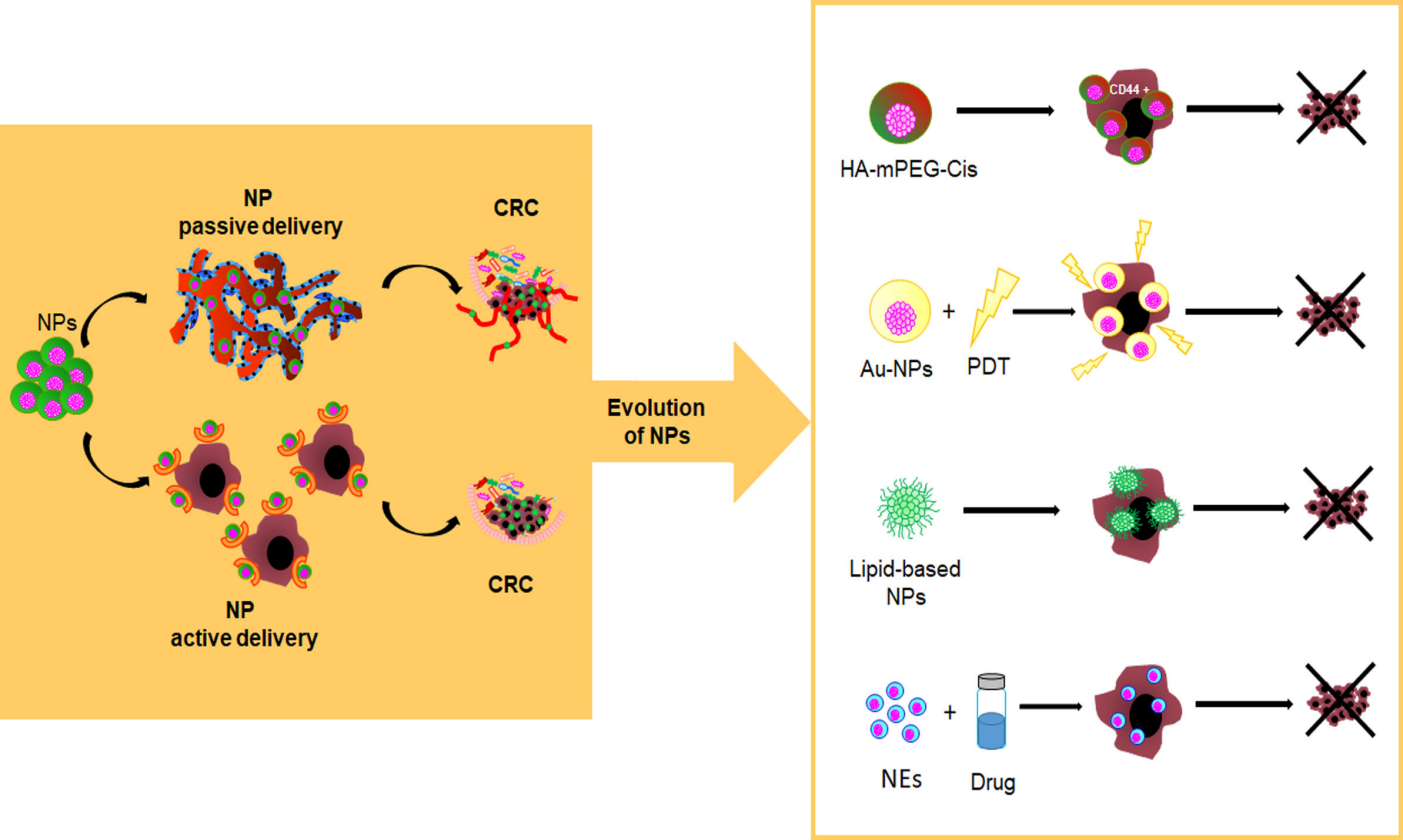
Evolution of nanoparticles as innovative drug delivery systems. pH-sensitive pegylated nano drug delivery systems (HA-mPEG-Cis NPs) are able to target CD44+ cells; Gold nanoparticles (AuNPs) find application in photodynamic therapy (PDT); Lipid-based NPs; Nanoemulsions (NEs) are a system to deliver hydrophobic drugs and hydrophilic or hydrophobic compounds.

Recently, it has been reported that NPs can accumulate in tumor tissues by passive or active delivery. Passive delivery of drug-loaded nanoparticles (i.e., the Enhanced Permeability and Retention, EPR, effect) is mainly due to fenestrated and immature new tumor vessels (128–131) while, the active delivery is due to a ligand-binding mechanism (e.g., nanoparticles targeting EpCAM, the folate receptor, EGFR and CD44) (132–134) (**Figure 3**). However, regardless of NP delivery, nanoparticle-protein complex, namely protein corona (PC), can permanently change the NP fate. The protein profile of the corona complex does not have a standard composition, because it varies not only among NPs of different chemical designs but even across the NPs of the same type. This latter is explained by the so-called Vroman effect, in which protein turnover depends not only by the high-affinity binding of proteins, but also on their exchange kinetics (135). In general, on the basis of the binding affinity between plasma proteins and NP surfaces a “hard corona” and a “soft corona” are distinguished, respectively (136). Moreover, proteins participating in the complex influence the cell recognition pathway by the reticuloendothelial system (RES) and promote biological processes against NPs, including aggregation, opsonization, and phagocytosis. Therefore, either second-generation NPs or PEGylation technique enhance the effect of cancer therapy by ensuring drug delivery within the tumor and evading phagocytosis (137). In this regard, emerging self-assembly pH-sensitive pegylated nano drug delivery systems, namely HA-mPEG-Cis NPs, are able to target CD44-CRC-positive cells and dissolve the hydrated PEG in the acidic tumor environment. These drug delivery systems improve drug circulation time and tumor targeting while reducing the side effects of the loaded drug (138) (**Figure 3**).

Inorganic nanocarrier, of controlled size and shape, such as gold NPs (AuNPs), show a certain versatility of use, including chemical sensing, imaging, and drug delivery due to their favourable optical and physical properties coupled with a reasonable biocompatibility with regard to biological environment (139). Interestingly, AuNPs, including gold nanorods, nanocages, nanostars, nanocubes, and nanospheres, find application in photodynamic therapy (PDT) for their specific physical features (i.e., optical and Surface Plasmon Resonance properties, proton-capture cross-section) (140) (**Figure 3**).

Lipid-based NPs are already FDA-approved for various therapeutic purposes, including cancer treatment (e.g., Doxil^®^, DaunoXome^®^, Myocet^®^, DepoCyt^®^, Marqibo^®^ and Onivyde^®^), severe infections or immunocompromised conditions (e.g. AmBisome^®^) and RNAi therapeutic (Onpattro^®^) (127, 141) (**Figure 3**).

Liposomes are small-size vesicles consisting of an outer lipid bilayer, synthetic or natural, and an aqueous core, widely used to encapsulate/entrap drugs or nucleic acids (i.e. gene therapy) (142). Currently, the manipulation of liposome lipid membrane components (e.g., neutral and/or negatively charged lipids plus cholesterol, sphingomyelin plus cholesterol, hydrogenated soy phosphatidylcholine plus cholesterol) as well as specific key parameters (e.g., size and shape) has improved their biological performance, in term of enhanced delivery efficiency, maximizing so-called nano-bio interactions (143).

Nanoemulsions (NEs) are another system to deliver hydrophobic drugs and hydrophilic or hydrophobic compounds through different routes of administration (e.g., aerosols, ingestion, and injections). NEs are made as single (i.e., oil-in-water [o/w], water-in-oil [w/o]) or dual (w/o/w, o/w/o) emulsions with biocompatible and FDA-approved biodegradable oils (143). Previous *in vitro* studies have shown that natural active compounds encapsulated within NEs, acting synergistically with chemotherapy, can improve the therapeutic value of treatment despite the use of a lower dosage of drug (144, 145). Also, the entrapment of active or cytotoxic drugs within nanoemulsions can be useful to sensitize CSCs to apoptosis (146) (**Figure 3**).

Over the past decade, nanotechnology has been widely explored to develop cytotoxic drug carriers. Although further improvements are needed, different types of NPs are already considered reliable systems for drug delivery due to their ability in targeting the tumor before releasing the drug.

## 4 Conclusion

Considering the critical nature of this review, and the variety of the included studies, it highlights that the sporadic CRC is a multi-stage and multi-step process in which the early mutational events seem to be driven by dysbiosis, chronic inflammation, and ROS. Moreover, treatments with standard cytotoxic agents, such as FOLFOX and FOLFIRI regimens, also contribute to the variation in the molecular profile of CRC in the advanced stage. Furthermore, this review also highlights that the limitation in treatment approaches for advanced CRC patients is mainly represented by both extrinsic (chemotherapy) and intrinsic mutation burden in cancer subpopulations (CSCs) developing MDR phenotype. In this regard, many strategies have been studied to overcome this issue, including the inhibition of crucial signaling involved in the self-renewal and metabolism of CSCs, as well as the redox-targeting approach. Moreover, using anti-vasculature therapies (e.g., bevacizumab and cetuximab) to modulate the tumor microenvironment represents a valid approach for enhancing cytotoxic drug uptake. Lastly, the development of novel DDS and promoter drugs can improve the delivery and the effectiveness of anti-cancer agents, opening up to personalized treatment protocols for CRC.

## Author contributions

Conceptualization: SV. Investigation: SV, ST. Writing – original draft: SV, ST, VA. Supervision: SV, AMB, CD, BM. Writing – review & editing: SV, BM, CD. All authors contributed to the article and approved the submitted version.
